# Effects of Microbiota-Driven Therapy on Circulating Trimethylamine-N-Oxide Metabolism: A Systematic Review and Meta-Analysis

**DOI:** 10.3389/fcvm.2021.710567

**Published:** 2021-09-06

**Authors:** Lina Miao, Jianpeng Du, Zhuhong Chen, Dazhuo Shi, Hua Qu

**Affiliations:** ^1^Department of Graduate School, Beijing University of Chinese Medicine, Beijing, China; ^2^Xiyuan Hospital, China Academy of Chinese Medical Sciences, Beijing, China; ^3^National Clinical Research Center for Chinese Medicine Cardiology, Beijing, China; ^4^NMPA Key Laboratory for Clinical Research and Evaluation of Traditional Chinese Medicine, Beijing, China

**Keywords:** synbiotics, prebiotics, probiotics, TMAO associated metabolites, meta-analysis

## Abstract

**Aim:** This study was designed to systematically evaluate the effects of microbiota-driven therapy on decreasing TMAO and its related metabolites.

**Methods and Results:** PubMed, EMBASE and Cochrane Library databases were searched (up to July 2021). Randomized controlled trials (RCTs), compared microbiota-driven therapy (prebiotics, probiotics, or synbiotics) with placebo on decreasing TMAO and its related metabolites, were eligible. Two researchers extracted the data independently and the disagreement was resolved by a third researcher. The risk of bias of included study was evaluated using Cochrane tool (RoB 2.0). Meta-analysis, meta-regression analysis and publication bias analysis were performed by RevMan 5.3 or Stata 12.0 software. Ten studies (12 arms) involving 342 patients (168 patients in the intervention group and 174 patients in the control group) were included. Compared with the control group, microbiota-driven therapy did not reduce circulating TMAO [SMD = −0.05, 95% CI (−0.36, 0.26), *P* = 0.749], choline [SMD = −0.34, 95% CI (−1.09, 0.41), *P* = 0.373], betaine aldehyde [SMD = −0.704, 95% CI (−1.789, 0.382), *P* = 0.204], and L-carnatine [SMD = −0.06, 95% CI (−0.38, 0.25), *P* = 0.692].

**Conclusion:** Current evidence does not support that microbiota-driven treatment reduce circulating levels of TMAO, choline, betaine aldehyde, and L-carnitine. However, given the small sample size, this conclusion needs to be proved in the future.

**Systematic Review Registration:** PROSPERO:CRD42019119107.

## Introduction

Trimethylamine-N-oxide (TMAO), a small organic compound with a colorless needle-like crystalline structure and a chemical formula of (CH3)3NO, plays an important role in stabilizing protein structure, regulating osmotic pressure and maintaining ion stability (1). Gut microbiota can integrate various dietary nutrients such as phosphatidylcholine/choline (2), L-carnitine (3), betaine, choline (4), dimethylglycine, and ergothioneine are metabolized to generate trimethylamine (TMA). Choline and L-carnitine are the most important precursors of TMAO. The serum TMAO concentration will increase after intake of dietary choline and L-carnitine. Most of the TMA is transported to the liver through the circulation, where it is oxidized by hepatic riboflavin monooxidase (FMO) to generate trimethylamine oxide (TMAO), and the excessive material is directly decomposed into dimethylamine (DMA) or methane (5). The host FMO family contains 5 functional enzymes (FMO1~FMO5), and FMO3 is the key rate-limiting enzyme for its high conversion efficiency of oxidizing TMA to TMAO (6). Fish and seafood are the direct sources of TMAO (7, 8). In addition, red meat, eggs and other dairy products are also the source of TMAO (9, 10). Although TMAO plays an important role in maintaining normal physiological activities of the human body, high TMAO level may lead to various diseases including cardiovascular events.

Accumulated evidence suggests that TMAO is a new marker of increased cardiovascular disease risk in humans. Tang et al. (11) conducted a 3-year follow-up of more than 4,000 patients who underwent selective coronary angiography. Their results illustrated that moderate (3.67–6.18 μM) and high (more than 6.18 μM) plasma TMAO levels were associated with major adverse cardiovascular events after excluding the effects of traditional cardiovascular risk factors such as LDL-C and C-reactive protein (HR, 1.88; 95% confidence interval, 1.44–2.44; *P* < 0.001). The highest quartile of plasma TMAO level was linked to a 2.5-fold higher risk of major cardiovascular events than the lowest quartile (quartile 1, <2.43 μM; quartile 4, more than 6.18 μM). Another result from a meta-analysis, which included 17 clinical studies (12) (26,167 subjects) with a mean follow-up time of 4.3 ± 1.5 years showed that high plasma TMAO level was associated with all-cause mortality and incidence of major cardiovascular and cerebrovascular events (*P* < 0.0001); in addition, a dose-effect relationship was observed between TMAO level and all-cause mortality (for every 10 μmol/L increase in TMAO, the relative risk of all-cause mortality went up by 7.6%). Further mechanistic research found that TMAO can increase the reactivity of platelets, thereby elevating the risk of thrombosis (13). Thus, finding drugs targeting the metabolic pathway of choline/TMA/TMAO and lower circulating TMAO level, thereby reducing platelet hyperreactivity, may be crucial for improving the prognosis of cardiovascular disease.

At present, drugs targeting the bacteria metabolic pathway of choline/TMA/TMAO in animal experiments include 3,3-dimethyl-1-butanol (14), the choline analogs IMC and FMC (15), and betaine aldehyde (16). However, those drugs have not been used in clinical research because of safety concerns. The production of TMAO needs the driving force of microbiota, and the method of changing the composition of intestinal microbiota is considered to have the potential in reducing TMAO level. In order to decrease TMAO level, the main intervention applied in clinical trials is microbiota-driven therapy, which includes probiotics, prebiotics and synbiotics. Ninety individuals with cardiovascular risk factors were included in the study of Tenore et al. and treated with probiotic for 8 weeks. The results demonstrated that microbiota-driven therapy could dramatically decreased the plasma TMAO level (17). Boutagy et al. observed the effects of probiotic on 19 healthy, non-obese males for 4 weeks, and found that probiotic treatment does not decrease plasma TMAO concentration (18). Therefore, the present meta-analysis was conducted to summarize the existing clinical studies and clarify the effects of microbiota-driven therapy on TMAO and its related metabolites in blood.

## Methods

This review was performed according to PRISMA (the preferred reporting item for systematic review and meta-analysis) (19). This systematic review and meta-analysis has been registered in PROSPERO as CRD42019119107.

### Search Strategies

Two reviewers (HQ and LM) independently searched PubMed, EMBASE and Cochrane Library for all potential literature till July 2021. All studies were restricted to be conducted in humans. The subjects consisted of patients who received microbiota-driven therapy (experimental group) or a placebo (control group). There were no limitations on age, gender, race or occupation. The terms “prebiotic,” “trimethylamine,” “trimethylamine N-oxide,” “TMA,” “TMAO,” “choline,” “betaine,” “L-carnitine,” “probiotic,” “synbiotic,” “dried yeast,” and “lactobacillus,” were searched for as key or free text words. Two reviewers (HQ and LM) separately searched the literatures and a third investigator (DS) was consulted to resolve any disputes. The search strategy is presented in Supplement 1.

### Study Selection

The studies were selected by two independent reviewers (LM and HQ) and approved by the third reviewer (SD). The studies were eligible if they met the following inclusion criteria: (1) randomized controlled trials (RCTs) with complete data available from the literature; (2) the experimental group received microbiota-driven therapy (probiotics/prebiotics/synbiotics), and the control group received the placebo; (3) the outcome included circulating TMAO level. The primary outcome was circulating TMAO level, and the secondary outcome included circulating TMA, choline, l-carnitine and betaine levels.

### Data Extraction

Relevant data were extracted from each individual eligible study using a structured table. Two reviewers (HQ and LM) extracted data independently and checked with each other. We contacted the authors to obtain the original data if the article lacked key information related to the investigation. Disagreement between the reviewers was resolved by consulting a third investigator (DS). Basic information and research content were extracted from each study. The information included the first author, publication year, duration, gender, age, sample size, participants, BMI and outcomes.

### Quality Assessment

Two authors (LM and HQ) independently assessed the risk of bias in the included studies according to the Cochrane risk-of-bias tool for randomized trials (RoB 2.0) (20). The RoB 2.0 tool evaluates the quality of literature from the aspects of randomization process, deviations from the intended interventions, missing outcome data, measurement of the outcome and selection of the reported result. Each item was estimated for risk of bias. The results from assessment were divided into three categories: (1) “low risk of bias”—all fields have tiny risk of bias; (2) “some problems”—at least one of area was identified as “causing some problems,” but was not a high risk of bias for any single area; (3) “high risk of bias”—at least one of domains showed high risk of bias or multiple domains produced “some problems.”

### Data Synthesis and Statistical Analysis

The data were analyzed with Stata (version 12.0) or Cochrane Collaboration software (RevMan 5.3) (21). Continuous variables were analyzed with the standard mean difference (SMD), and effect sizes were showed as 95% confidence intervals (CIs). Statistical heterogeneity was measured with *I*^2^ statistic. *I*^2^ ≤ 50% indicated that the heterogeneity was not significant among the studies, and fixed-effect models were applied. *I*^2^ > 50% indicated that the heterogeneity among the studies was statistically significant, random-effect models were applied. Meta-regression and subgroup analysis were performed to find the source of heterogeneity when the included studies ≥10. If necessary, subgroup analysis based on factors such as sex, age and intervention measures was conducted to clarify their impact on outcome. Sensitivity analysis was used to observe whether the results were reliable after the studies were excluded one by one. Publication bias was evaluated using funnel plots and egger's test. Meta-regression analysis was conducted for the factors that affected the research results, such as sex, age and duration of treatment, to observe their impact on outcomes.

## Results

### Literature Search and Screening

**Figure 1:**
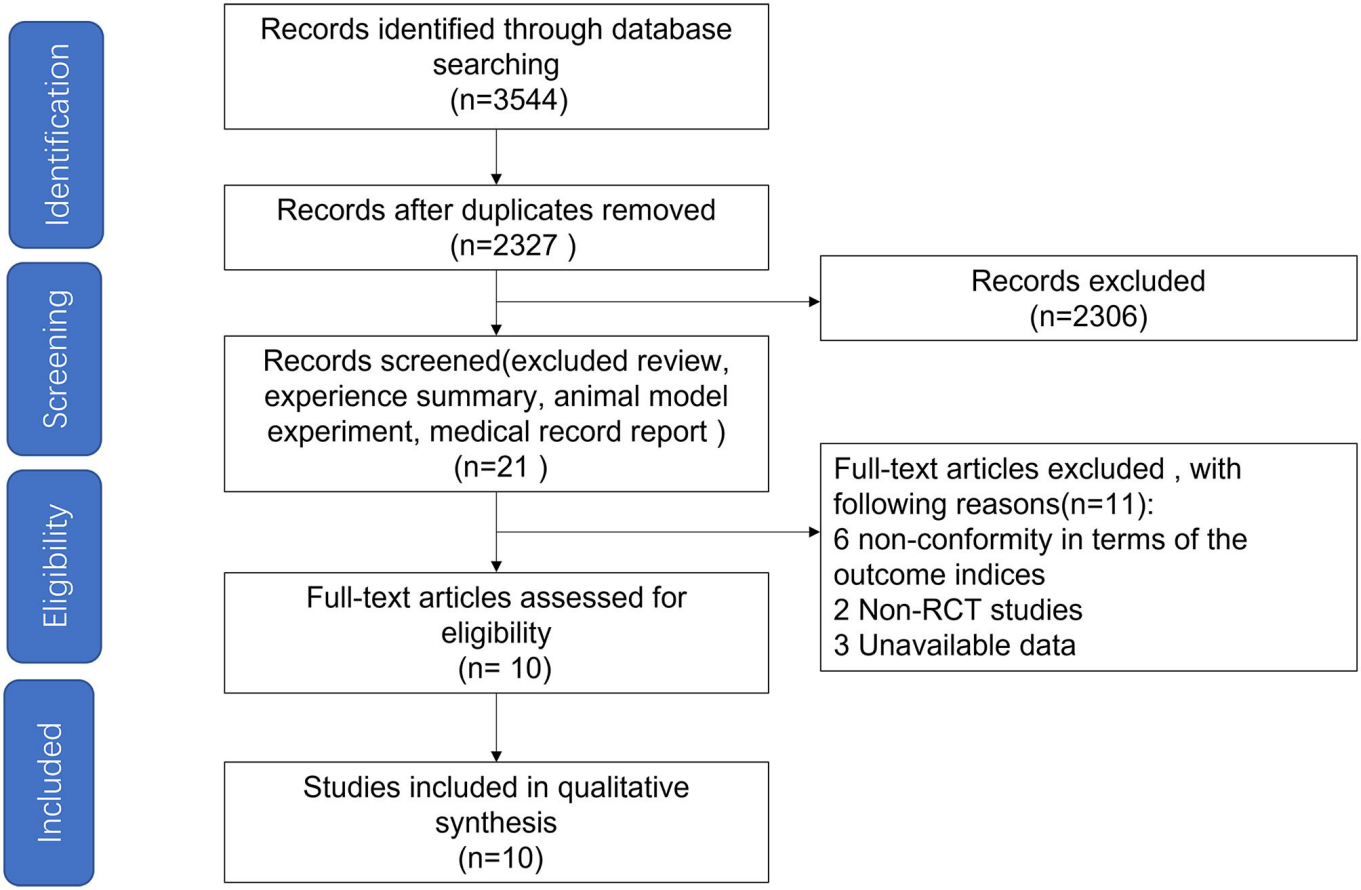
Flow diagram depicting the article search and selection strategy. A total of 10 articles were included in the study.

Based on the searching strategy, we identified 3,544 potential studies, after removing duplication (*n* = 1,217) and studies that didn't meet inclusion criteria by titles and abstracts (*n* = 2,306), 21 studies were included. The full texts of the remaining 21 studies were reviewed, and studies failing to meet the inclusion criteria, including no RCT design, unavailable data and studies non-conformity in terms of the outcome indices, were excluded. Finally, ten studies (12 arms) published in English met our inclusion criteria (Figure 1) (17, 18, 22–29).

### Study Characteristics

Ten studies included a total of 342 patients, including 168 patients in the microbiota-driven therapy group (including probiotics, prebiotics or synbiotics) and 174 patients in the control group. The duration of treatment ranged from 28 to 90 days among the studies. All included studies assessed the effect of microbiota-driven therapy on circulating TMAO level, and 4 studies (18, 22, 23, 27) observed the effect of microbiota-driven therapy on circulating choline level. Three studies (5 arms) (18, 23, 29) observed the effect of microbiota-driven therapy on circulating l-carnitine level, and 3 studies (18, 23, 27) observed the effect of microbiota-driven therapy on circulating betaine level. The basic information included in the study is presented in Table 1. All studies compared sex, age and other variables among the groups, and no significant differences were found.

**Table 1:** Characteristics of the trials included in this meta-analysis.

**References**	**Intervention**	**Duration**	**Individual (T/P)**	**Mean age (y) (T/C)**	**Male (%) (T/C)**	**Participants**	**BMI (kg/m^2^) (T/C)**	**Outcome**
Baugh et al. (23)	Prebiotic vs. placebo	42 days	18 (7/11)	58 ± 3/58 ± 3	28.6/45.5	Overweight or obese patients at risk for T2DM	30.8 ± 0.8/31.0 ± 0.92	TMAO→, L-carnitine↓, choline↓, betaine↑
Borges et al. (22)	Probiotic vs. placebo	90 days	21 (11/10)	54.0/54.0	40.0/27.3	Hemodialysis Patients	24.6 ±7.9/26.7 ± 14.2	TMAO→, choline↑, betaine↑
Boutagy et al. (18)	Probiotic vs. placebo	28 days	19 (9/10)	22.4 ± 61.1/22.5 ± 6 1.0	100.0/100.0	Non-obese college aged males	24.5 ± 1.1/23 ± 0.5	TMAO↑, L-carnitine↑, choline↑, betaine↑
Poesen et al. (24)	Prebiotic vs. placebo	28 days	80 (40/40)	36.7 ± 9.1/32.0 ± 2.0	33.3/0	CKD patients	28.7 ± 5.0/28.7 ± 5.0	TMAO↓
Tripolt et al. (25)	Probiotic vs. placebo	84 days	28 (13/15)	10.4 ± 0.3/10.2 ± 0.4	54.5/60.0	Adult metabolic syndrome patients	3 5 ± 5/3 2 ± 4	TMAO →
de Faria Barros et al. (27)	Probiotic vs. placebo	90 days	22 (12/10)	64.7 ± 6.7/63.6 ± 8.5	50.0/50.0	Non-dialysis CKD patients	26.7 ± 3.6/27.2 ± 3.9	TMAO↓, choline↑, betaine↑
Mafra et al. (26)	Probiotic vs. placebo	90 days	16 (7/9)	64.2 ± 7.2/63.4 ± 8.1	N/N	Non-dialysis CKD patients	–	TMAO↓
Tenore et al. (17)	Probiotic vs. placebo	56 days	54 (27/27)	48.2 ± 10.2/45.1 ± 10.3	55.5/55.5	Individuals with cardiovascular disease risk factors	–	TMAO↓
Dahl et al. (29)	Probiotic vs. placebo	28 days	20 (20/20)	72.4 ± 5.0/72.4 ± 5.0	0/0	Older healthy women	≤ 30	TMAO→, L-carnitine →
Dahl et al. (29)	Prebiotic vs. placebo	28 days	20 (20/20)	72.4 ± 5.0/72.4 ± 5.0	0/0	Older healthy women	≤ 30	TMAO→, L-carnitine↑
Dahl et al. (29)	Synbiotic vs. placebo	28 days	20(20/20)	72.4 ± 5.0/72.4 ± 5.0	0/0	Older healthy women	≤ 30	TMAO→, L-carnitine↑
Moludi et al. (28)	Probiotic vs. placebo	90 days	44 (22/22)	56.70 ± 9.10/57.10 ± 7.80	N/N	Patients with new MI who underwent PTCA	–	TMAO↓

*TMA, trimethylamine; TMAO, Trimethylamine-N-Oxide; PTCA, percutaneous transluminal coronary angioplasty; T, test group; C, control group;→, no significant change before and after intervention; ↑, increased after intervention; ↓, decreased after intervention*.

### Methodology Quality Evaluation of the Included Studies

**Figure 2:**
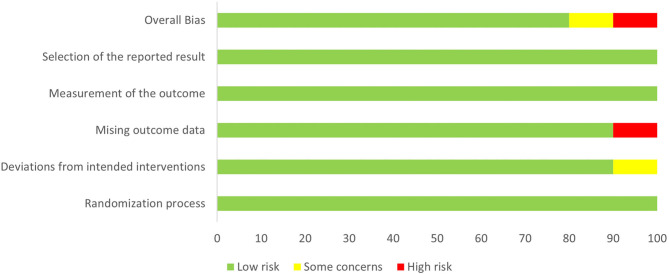
Literature quality evaluation (RoB 2.0).

**Figure 3:**
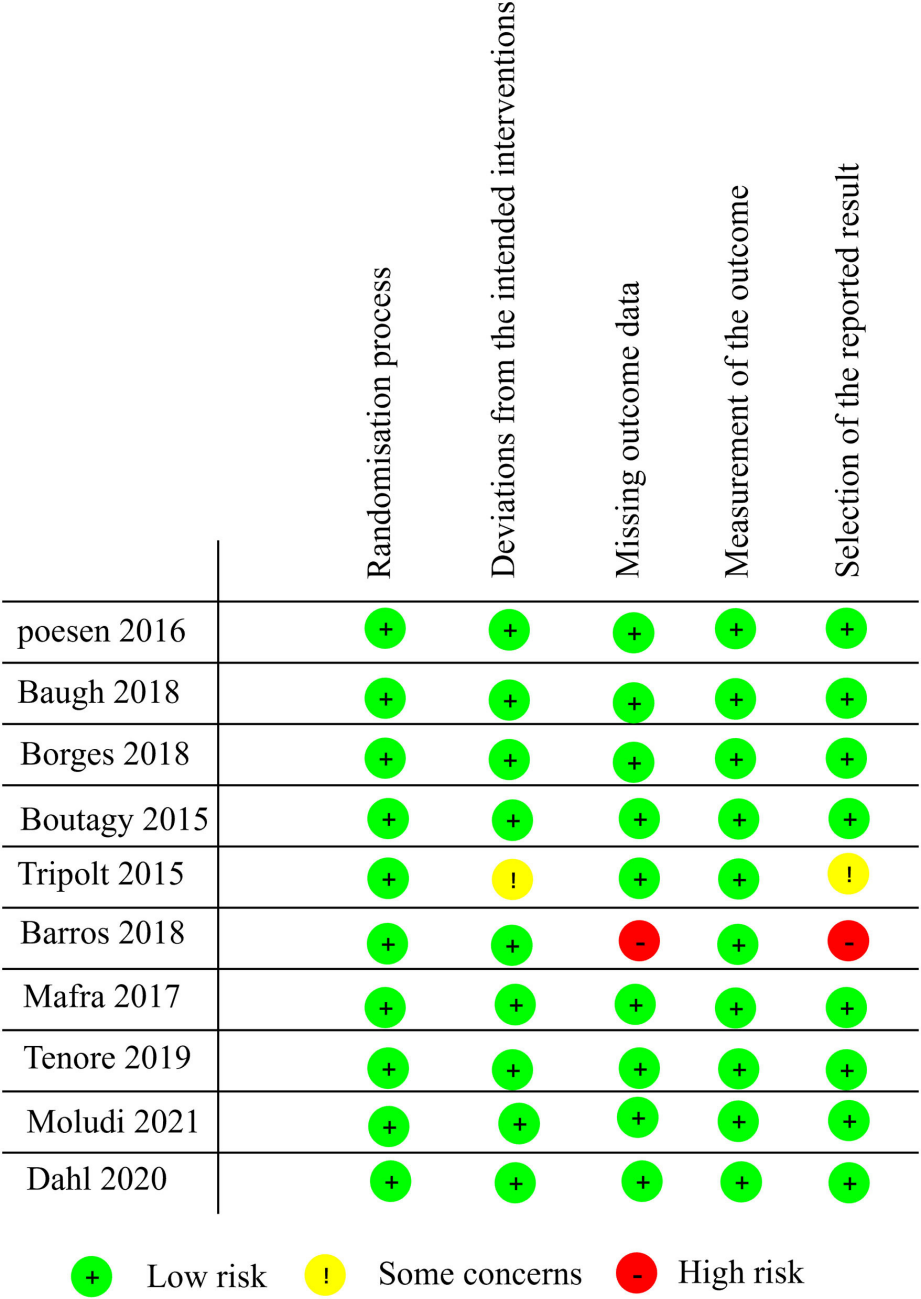
Summary of the quality evaluation of the literature (RoB 2.0).

Study quality was assessed according to RoB 2.0 tool (20), and 8 studies were assessed as “low risk of bias,” one was assessed as “some concerns” and one as “high risk of bias.” The quality evaluation of the literature is shown in Figures 2, 3.

### Meta-Analysis Results

#### Effect of Microbiota-Driven Therapy on Circulating TMAO Level

**Figure 4:**
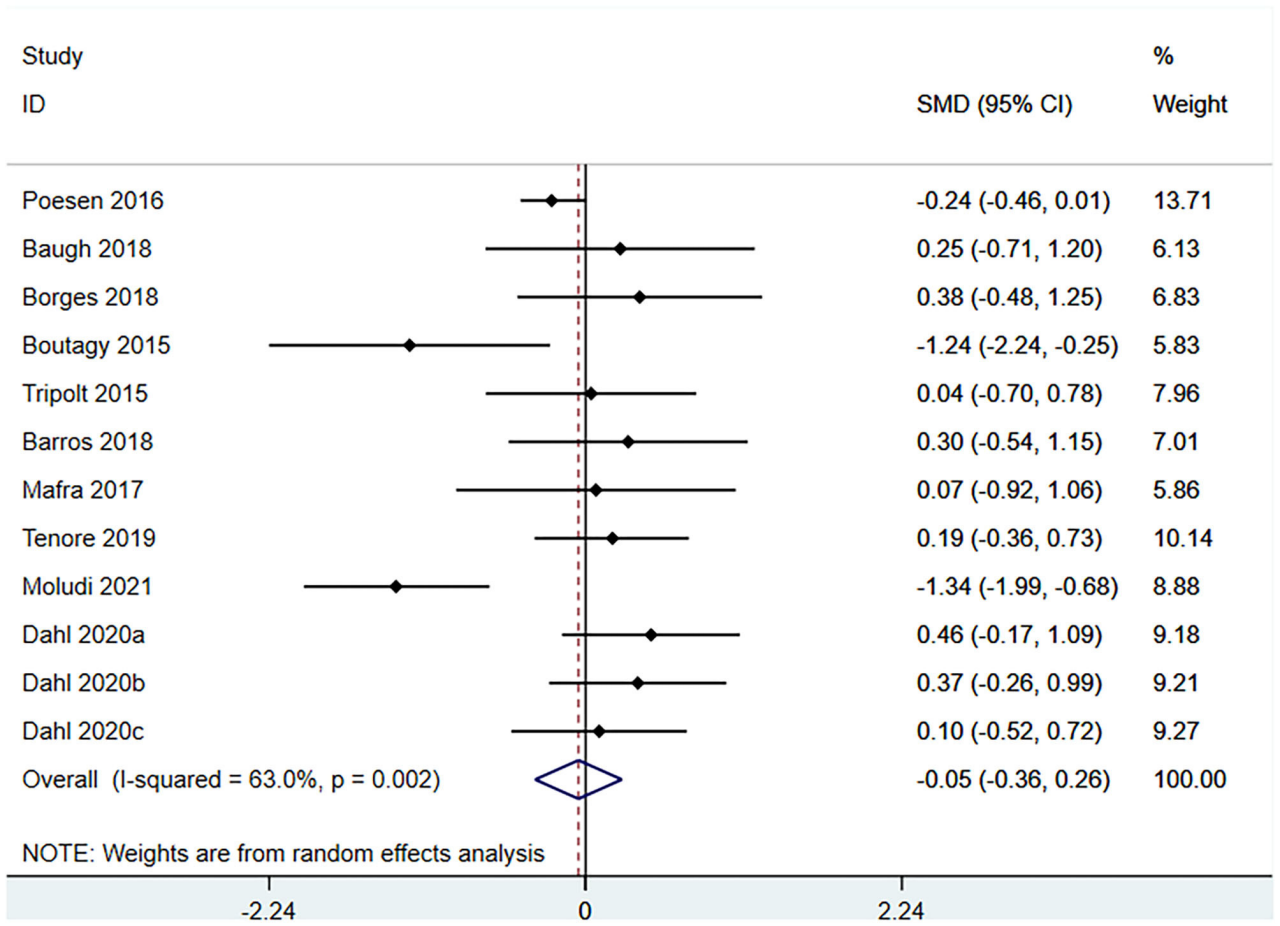
Forest plot for the effect of microbiota-driven therapy on circulating TMAO level. The effect size of each trial or the overall was represented as black diamond and the weight of each trail in the overall study was represented as gray box around black diamond. The estimated 95% confidence interval of effect size was identified by extending lines. CI, confidence interval; RCT, randomized controlled trial; SMD, Standard Mean Difference.

**Figure 5:**
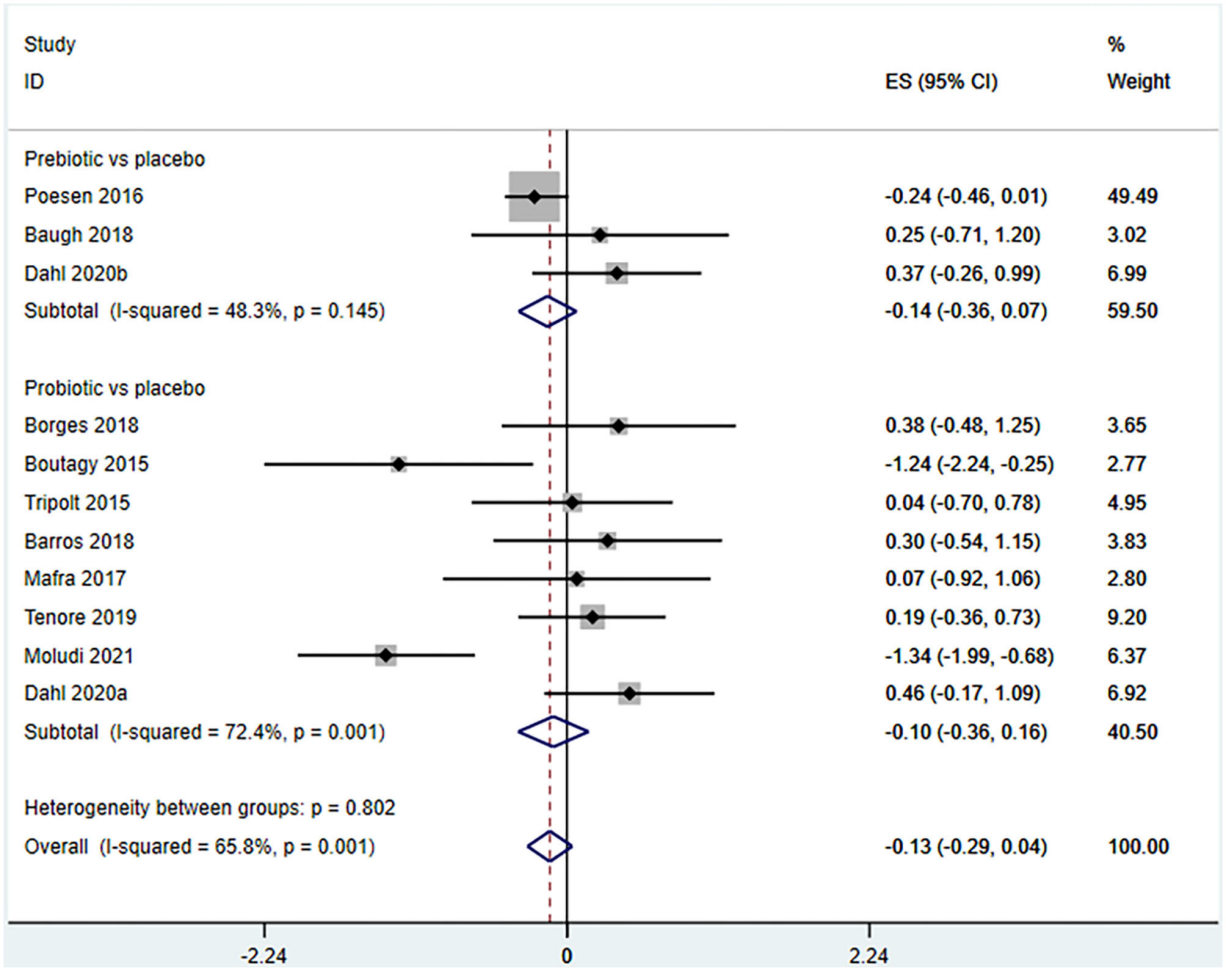
Forest plot for the effect of microbiota-driven therapy on circulating TMAO level (subgroup analysis). The effect size of each trial or the overall was represented as black diamond and the weight of each trail in the overall study was represented as gray box around black diamond. The estimated 95% confidence interval of effect size was identified by extending lines. CI, confidence interval; RCT, randomized controlled trial; SMD, Standard Mean Difference.

**Figure 6:**
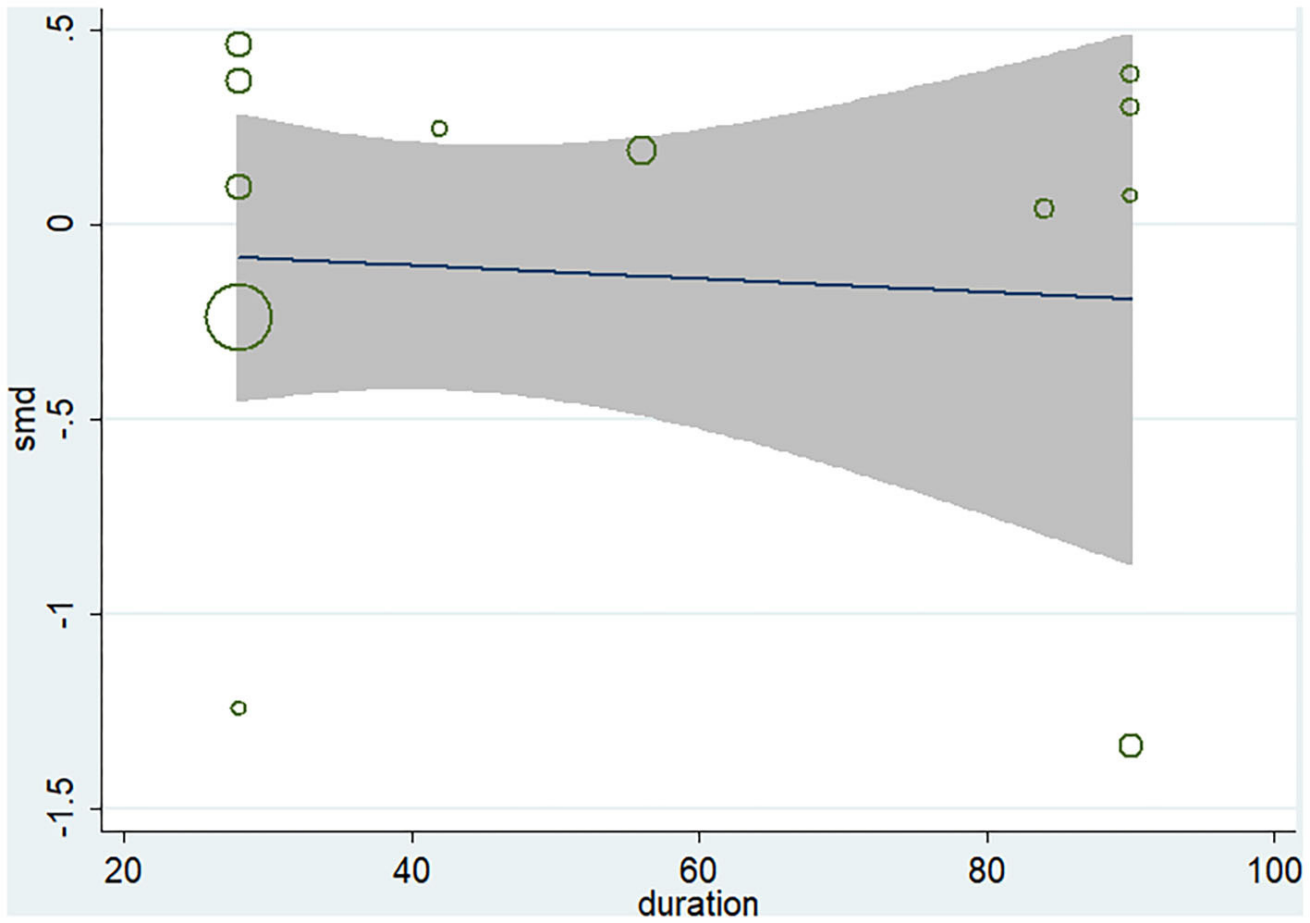
Meta-regression analysis of the influence of intervention time of microbiota-driven therapy on circulating TMAO.

Ten studies (12 arms) (17, 18, 22–29) observed the effect of microbiota-driven therapy on circulating TMAO level (Figure 4). There was an obvious heterogeneity among the included studies (*I*^2^ = 63.0%, *P* = 0.172), and a random-effect model was used to assess the effects of treatment. The results revealed no difference in circulating TMAO level between the intervention and control groups (SMD = −0.05, 95% CI = −0.36–0.26, *P* = 0.749). To explore the source of heterogeneity, we conducted a subgroup analysis. Subgroup analysis illustrated that prebiotics (SMD = 0.01, 95% CI = −0.43–0.45, *P* = 0.145) and probiotics (SMD = −0.13, 95% CI = −0.64–0.38, *P* = 0.001) did not reduce circulating TMAO level (Figure 5). Further meta-regression analysis showed that the intervention duration of microbiota-driven therapy was not related to the level of TMAO in circulation (*P* = 0.803) (Figure 6).

#### Effect of Microbiota-Driven Therapy on Circulating Choline Level

**Figure 7:**
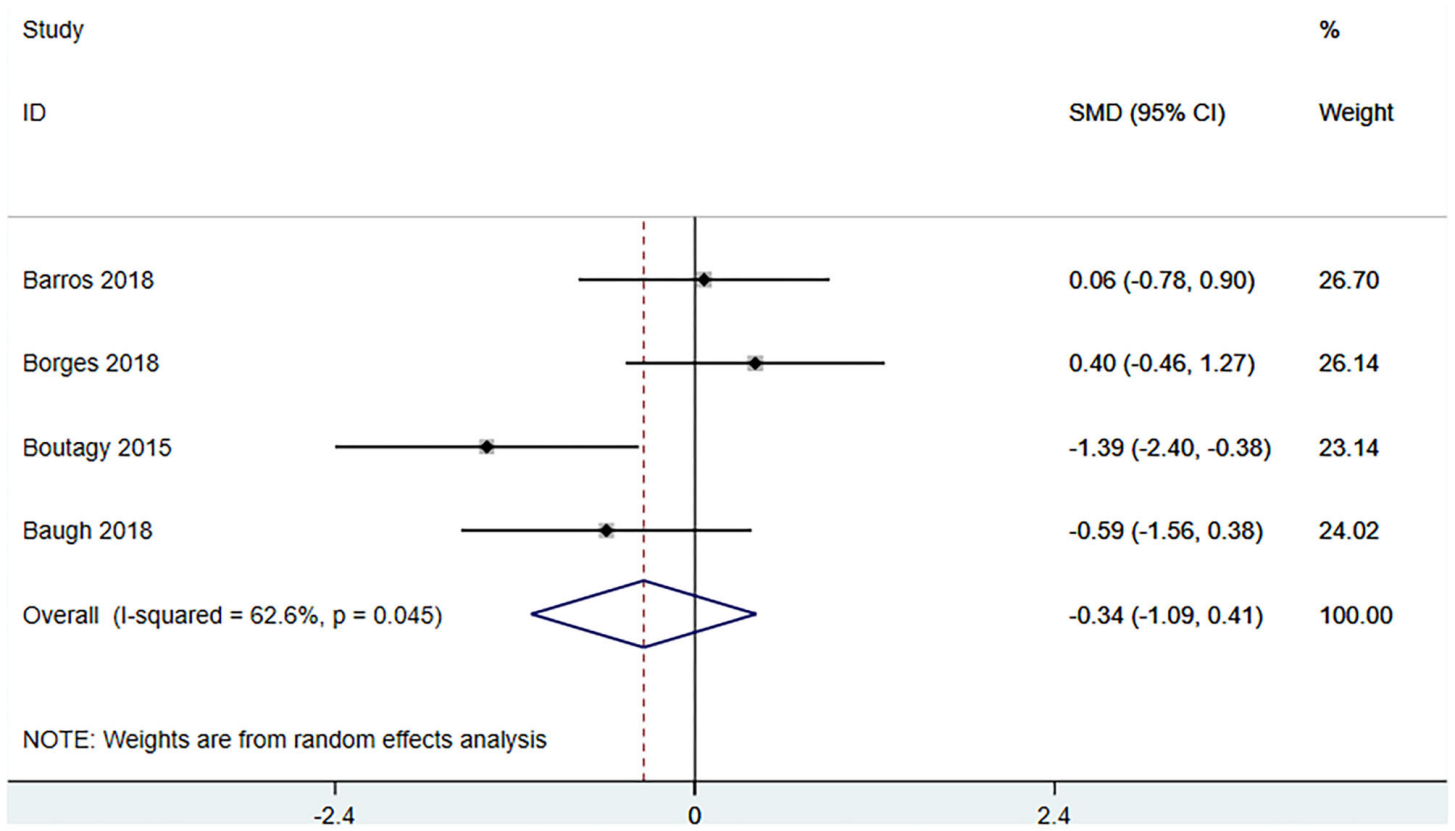
Forest plot for the effect of microbiota-driven therapy on circulating choline level. The effect size of each trial or the overall was represented as black diamond and the weight of each trail in the overall study was represented as gray box around black diamond. The estimated 95% confidence interval of effect size was identified by extending lines. CI, confidence interval; RCT, randomized controlled trial; SMD, Standard Mean Difference.

**Figure 8:**
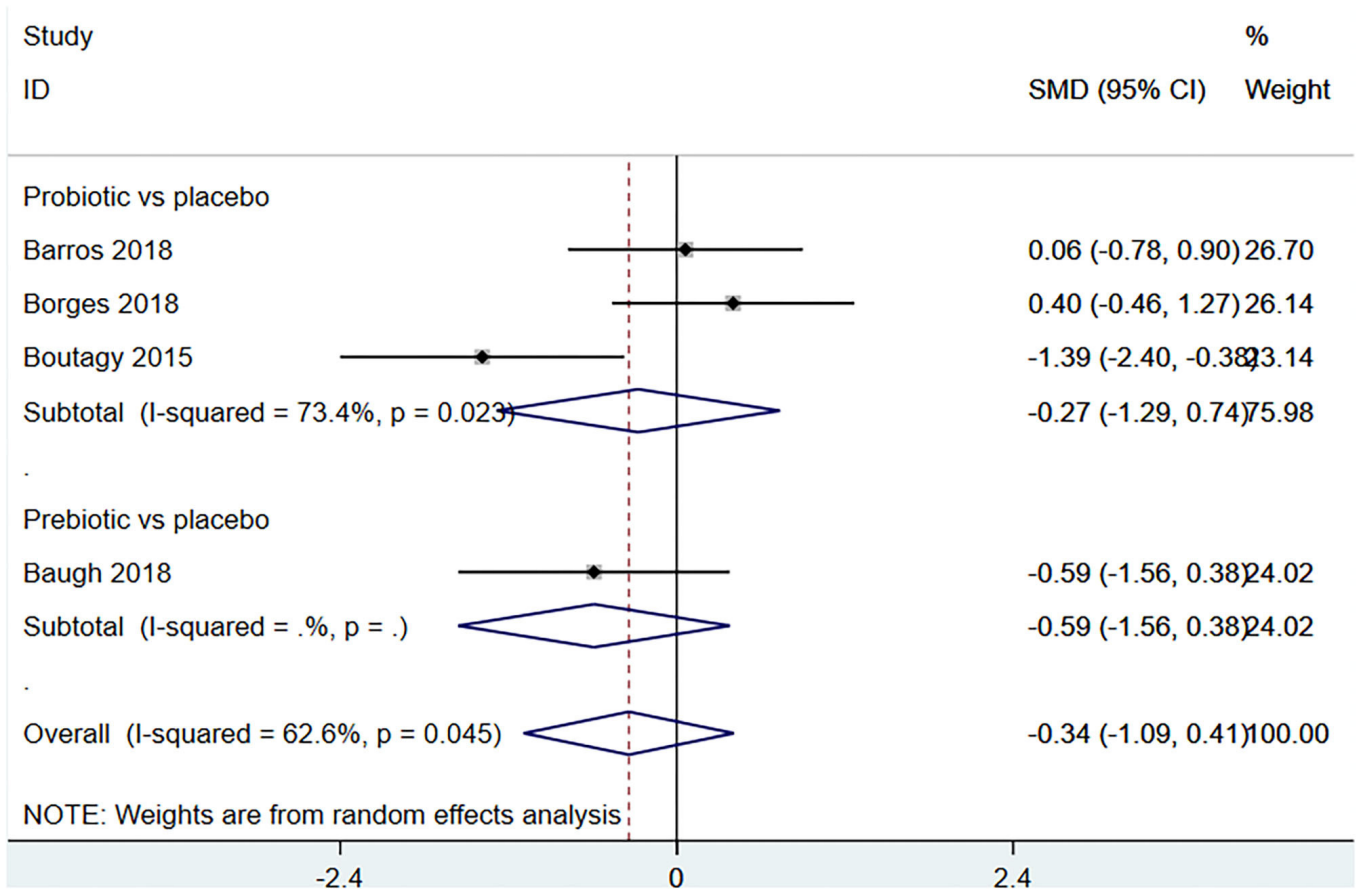
Forest plot for the effect of microbiota-driven therapy on circulating choline level (subgroup analysis). The effect size of each trial or the overall was represented as black diamond and the weight of each trail in the overall study was represented as gray box around black diamond. The estimated 95% confidence interval of effect size was identified by extending lines. CI, confidence interval; RCT, randomized controlled trial; SMD, Standard Mean Difference.

Four studies (18, 22, 23, 27) (including 80 patients) observed the effect of microbiota-driven therapy on circulating choline level (Figure 7). Obvious heterogeneity was observed among the included studies (*I*^2^ = 62.6%, *P* = 0.045). A random-effect model was used to assess the effects of therapy. The results revealed no significant difference in circulating choline level between the intervention and control groups (SMD = −0.34, 95% CI = −1.09–0.41, *P* = 0.373, Figure 7). Subgroup analysis revealed that prebiotics (SMD = −0.591, 95% CI = −1.561–0.378) and probiotics (SMD = −0.275, 95% CI = −1.287–0.737, *P* = 0.023) did not reduce circulating choline level (Figure 8).

#### Effect of Microbiota-Driven Therapy on Circulating Betaine Aldehyde Level

**Figure 9:**
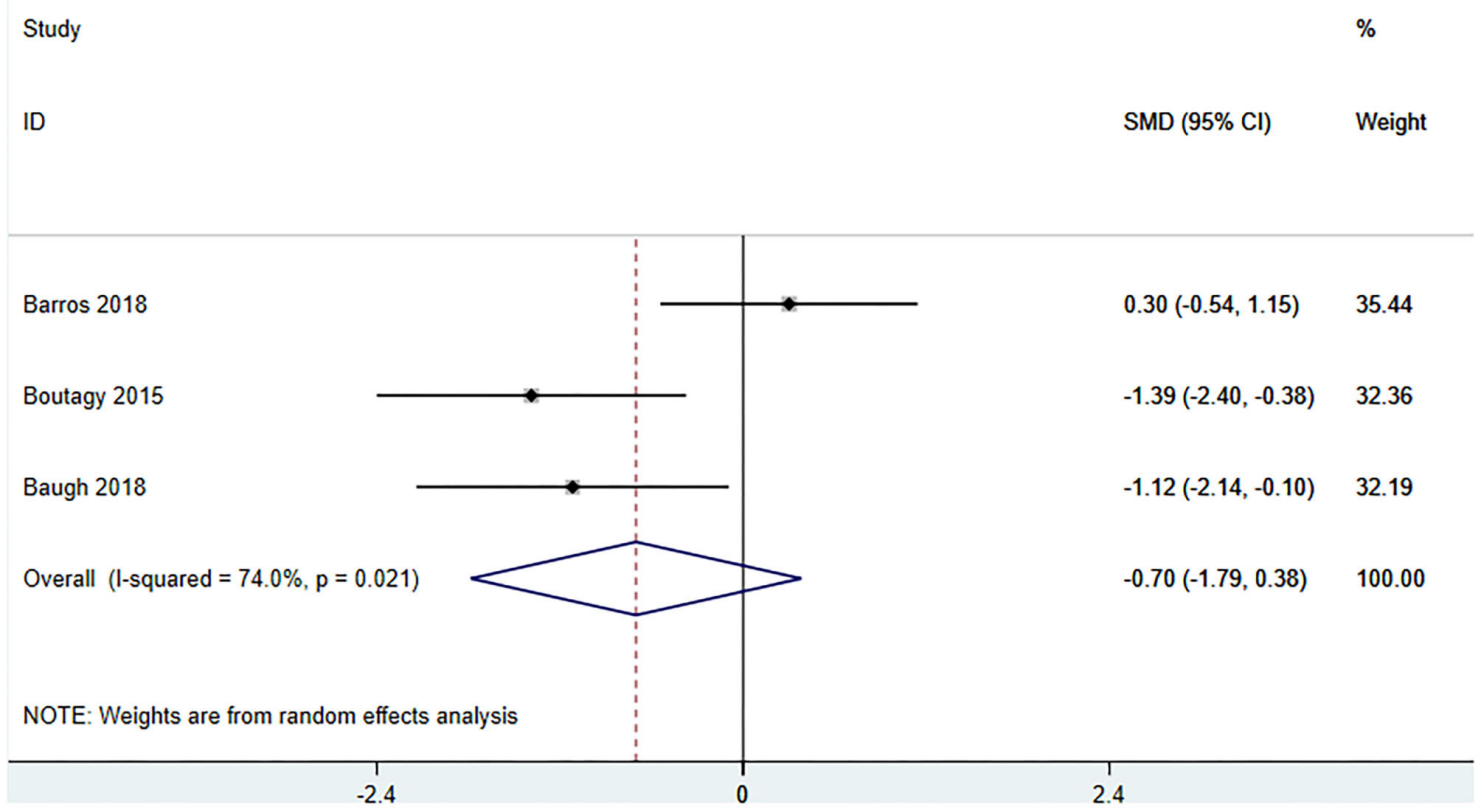
Forest plot for the effect of microbiota-driven therapy on circulating betaine aldehyde level. The effect size of each trial or the overall was represented as black diamond and the weight of each trail in the overall study was represented as gray box around black diamond. The estimated 95% confidence interval of effect size was identified by extending lines. CI, confidence interval; RCT, randomized controlled trial; SMD, Standard Mean Difference.

**Figure 10:**
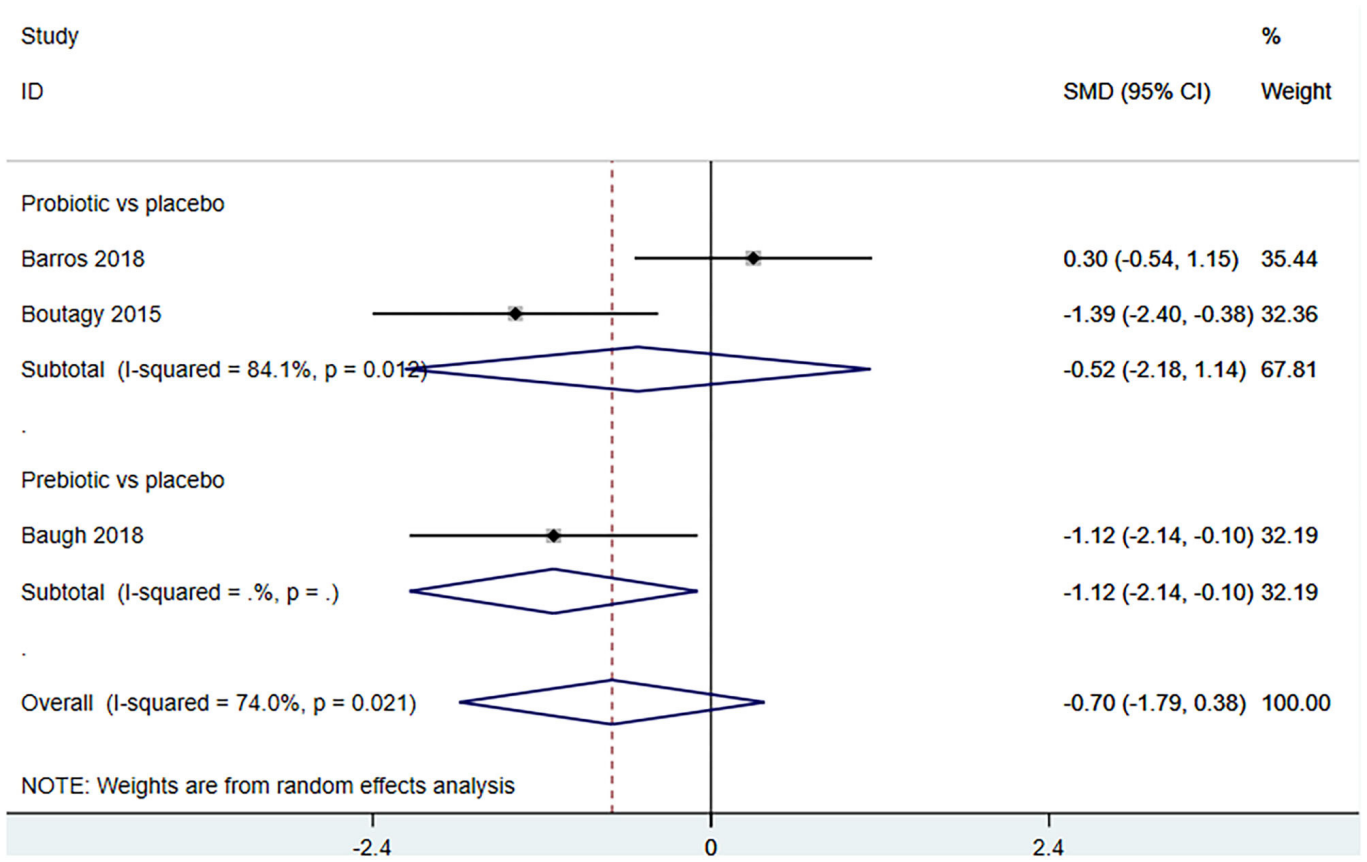
Forest plot for the effect of microbiota-driven therapy on circulating betaine aldehyde level (subgroup analysis). The effect size of each trial or the overall was represented as black diamond and the weight of each trail in the overall study was represented as gray box around black diamond. The estimated 95% confidence interval of effect size was identified by extending lines. CI, confidence interval; RCT, randomized controlled trial; SMD, Standard Mean Difference.

Three studies (18, 23, 27) (including 59 patients) investigated the effect of microbiota-driven therapy on betaine aldehyde level in circulation (Figure 9). The heterogeneity among the included studies was obvious (*I*^2^ = 74%, *P* = 0.021), and a random-effect model was used. The results revealed no significance difference in circulating betaine aldehyde level between the intervention and control groups (SMD = −0.704, 95% CI = −1.789–0.382, *P* = 0.204). Subgroup analysis indicated that probiotics did not reduce circulating betaine aldehyde level (SMD = −0.520, 95% CI = −2.177–1.137, *P* = 0.538) (Figure 10).

#### Effect of Microbiota-Driven Therapy on Circulating L-Carnitine Level

**Figure 11:**
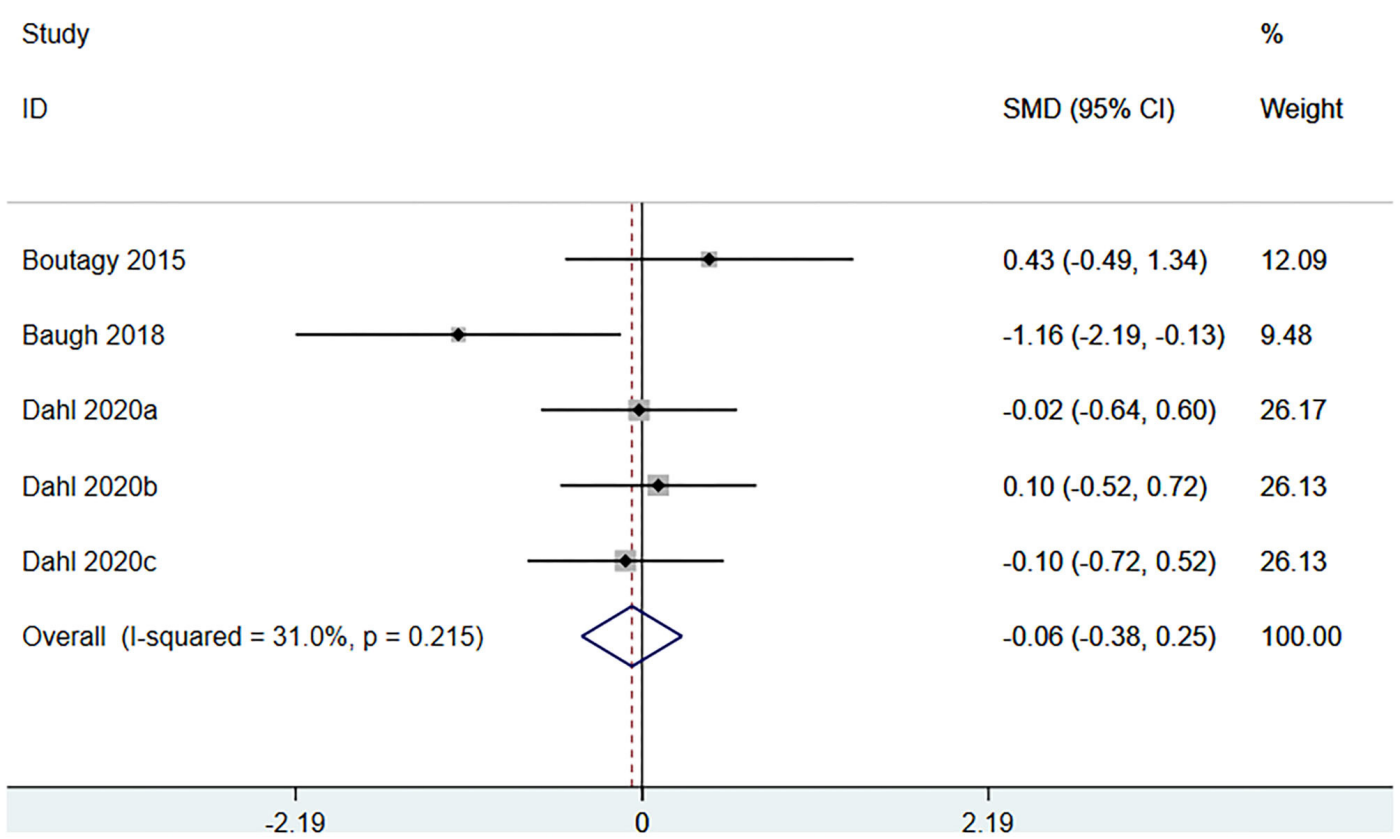
Forest plot for the effect of microbiota-driven therapy on circulating L-carnitine level. The effect size of each trial or the overall was represented as black diamond and the weight of each trail in the overall study was represented as gray box around black diamond. The estimated 95% confidence interval of effect size was identified by extending lines. CI, confidence interval; RCT, randomized controlled trial; SMD, Standard Mean Difference.

**Figure 12:**
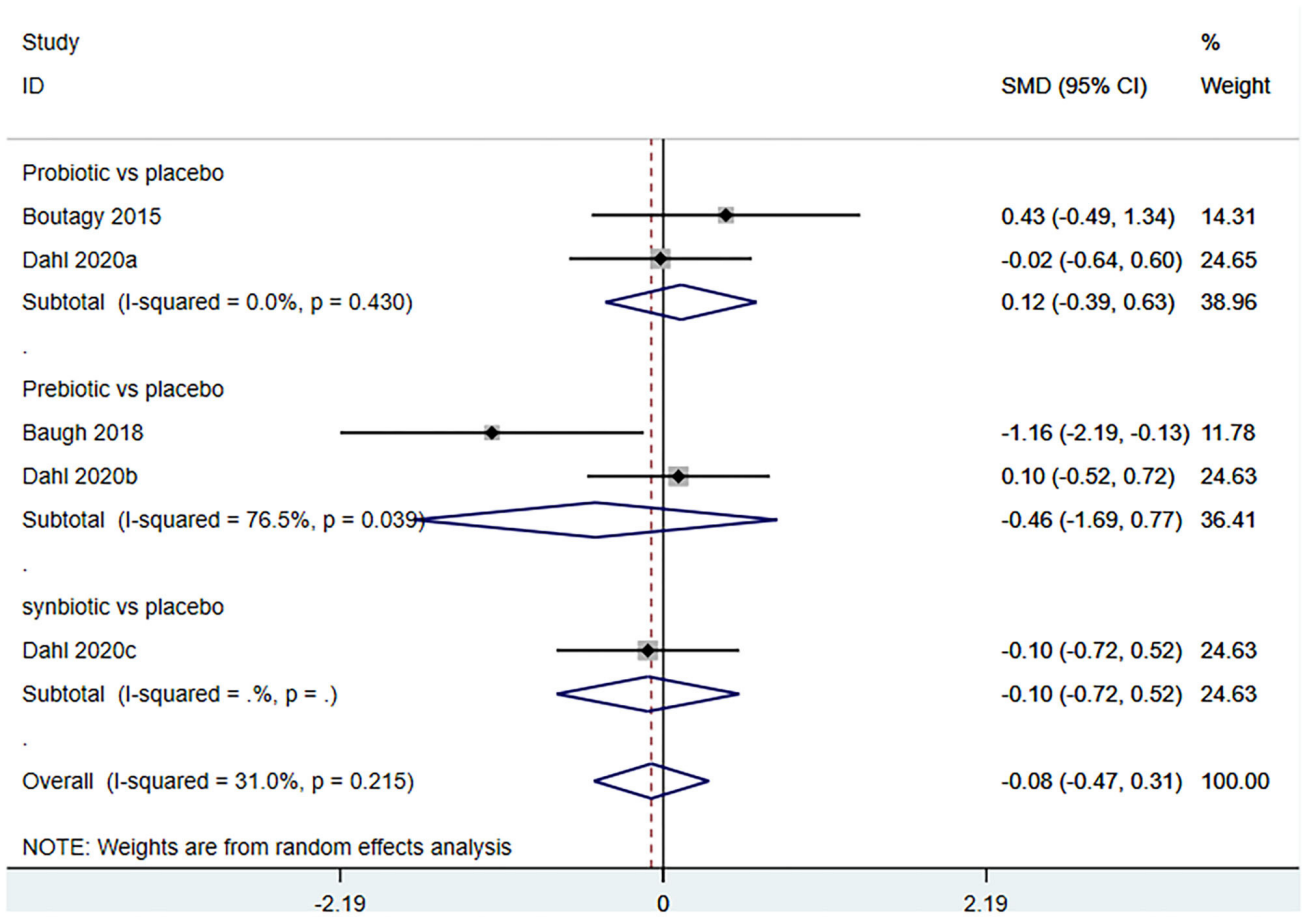
Forest plot for the effect of microbiota-driven therapy on circulating L-carnitine level (subgroup analysis). The effect size of each trial or the overall was represented as black diamond and the weight of each trail in the overall study was represented as gray box around black diamond. The estimated 95% confidence interval of effect size was identified by extending lines. CI, confidence interval; RCT, randomized controlled trial; SMD, Standard Mean Difference.

Three studies (5 arms) (18, 23, 29) (including 117 patients) assessed the effect of microbiota-driven therapy on circulating L-carnitine level (Figure 11). The heterogeneity among the included studies was not obvious (*I*^2^ = 31.0%, *P* = 0.215), and a fixed-effect model was used. The results revealed no statistical difference in circulating L-carnitine level between the intervention and control groups [SMD = −0.06, 95%CI (−0.38, 0.25), *P* = 0.692] (Figure 11). Subgroup analysis illustrated that prebiotics (SMD = −0.46, 95% CI = −1.69–0.77, *P* = 0.039) and probiotics (SMD = 0.12, 95% CI = −0.39–0.63, *P* = 0.430) did not reduce circulating L-carnitine level (Figure 12).

### Publication Bias Analysis

**Figure 13:**
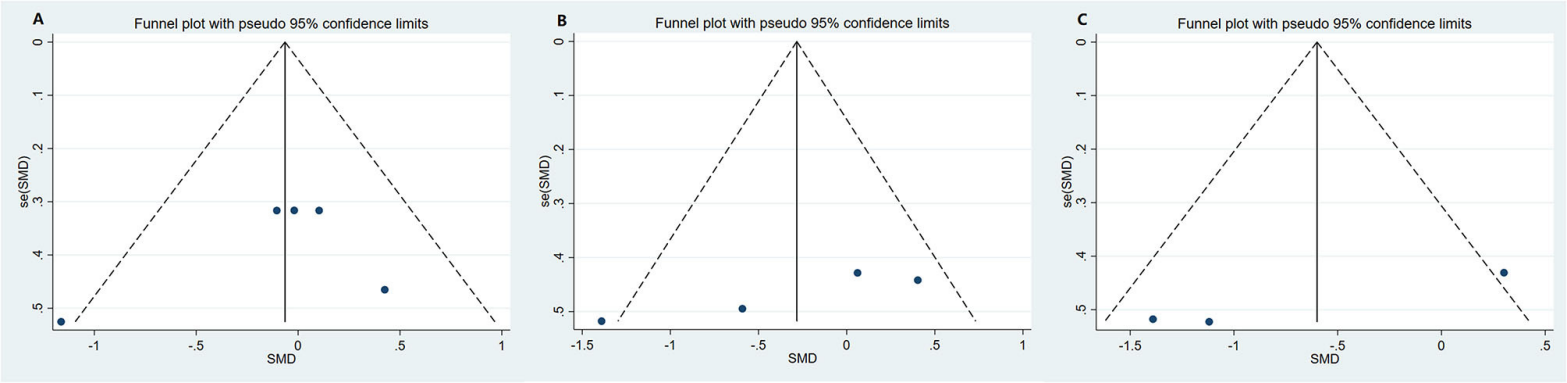
Funnel plot for publication bias of study. **(A)** Funnel plot for publication bias of the effect of microbiota-driven therapy on circulating choline level; **(B)** Funnel plot for publication bias of the effect of microbiota-driven therapy on circulating betaine aldehyde level; **(C)** Funnel plot for publication bias of the effect of microbiota-driven therapy on circulating L-carnitine level.

**Figure 14:**
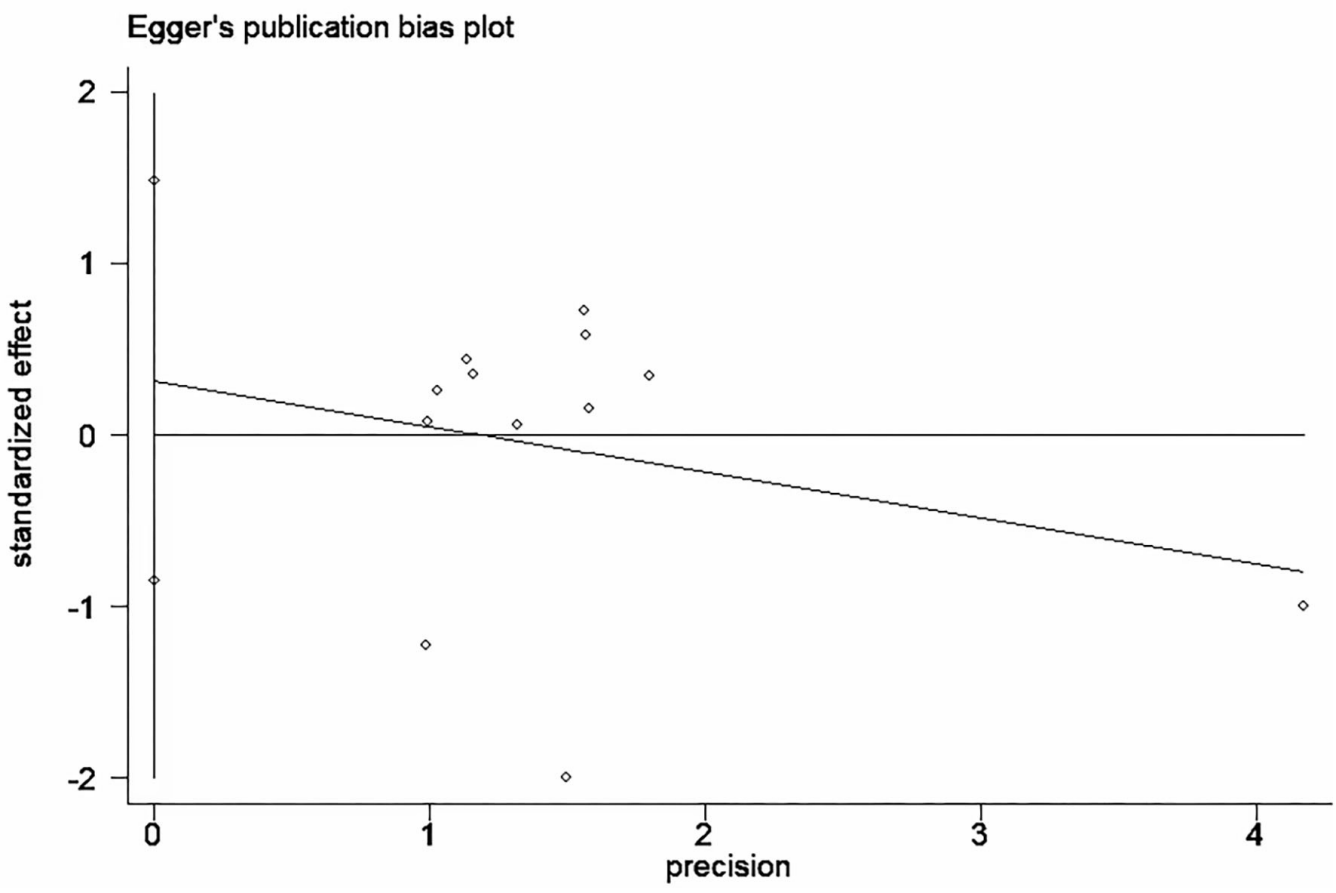
Published bias egger test for the effect of microbiota-driven therapy on circulating TMAO level.

Funnel plot was used to analyze the publication bias of the outcome. And there was no obvious publication bias according to funnel plot (Figures 13A–C). Egger's test was also used to analyze the publication bias of the outcome regarding TMAO level. No significant published bias was identified (Egger's regression, coefficient, −0.268, 95% CI = −0.927–0.391, *P* = 0.387) (Figure 14).

### Sensitivity Analysis

**Figure 15:**
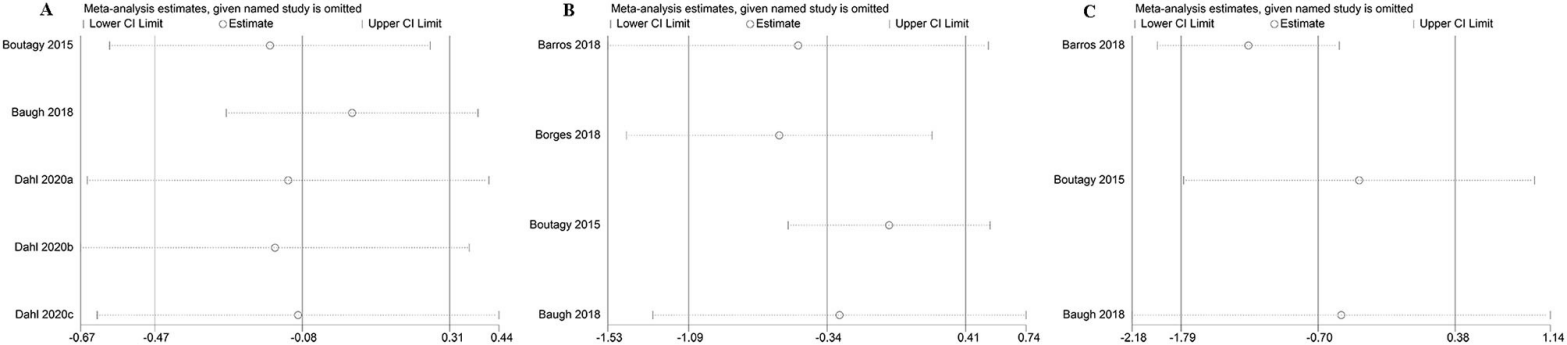
The sensitivity analysis of the study. **(A)** The sensitivity analysis of microbiota-driven therapy on circulating choline level; **(B)** The sensitivity analysis of microbiota-driven therapy on circulating betaine aldehyde level; **(C)** The sensitivity analysis of microbiota-driven therapy on circulating L-carnitine level.

The sensitivity analysis of the outcome with high heterogeneity (*I*^2^ ≥ 50) illustrated that the results were reliable after excluding the studies one by one. This indicated that despite the existence of the heterogeneity, but the effect size was still reliable (Figure 15).

## Discussion

Cardiovascular disease is a major disease endangering human health across the world (30), and the mortality of thrombus-related cardiovascular disease continues to increase. Therefore, it is necessary to find effective treatments which could reduce the risk of thrombotic events in patients with cardiovascular disease (31). TMAO, as a newly identified independent risk factor for cardiovascular events, is a promising target for preventing and treating cardiovascular events (11, 12). Early clinical studies with small samples found that microbiota-driven therapy may be an effective method to reduce TMAO level, but due to the small sample size, the research conclusions need further confirmation.

At present, the mechanism of TMAO increasing cardiovascular risk and death is not very clear. Wang et al. (32) reported that TMAO promoted the deposition of cholesterol in peripheral cells (for example those in the arterial wall), and cholesterol removal decreased at the same time. Hardin et al. (33) described the possible mechanism that linking TMAO and atherosclerotic factor is that the increase in plasma TMAO concentration promoted the upregulation of macrophage scavenger receptors, which contributed to the recruitment of macrophages and consequently inflammation. Atherosclerosis is aroused by corresponding inflammation, macrophages accumulation and foam cells generation. Leustean et al. (34) described other effects of TMAO in CVDs, including increased platelet aggregation and reduced nitric oxide level.

Fish and seafood are direct dietary sources of TMAO (7, 8). A variety of dietary nutrients, such as phosphatidylcholine/choline, L-carnitine, betaine, choline, dimethyl glycine and thioneine are important precursors for the synthesis of TMAO. The production of TMAO requires the action of gut microbes (35). Without catalyzing of gut microbes, humans and mice will not be able to produce TMA, and the liver needs TMA to make TMAO (11). Therefore, regulating the gut microbes is considered a promising way to regulate the level of TMAO. It has been found that the composition of gut microbiota is related to atherosclerosis, thrombosis and chronic heart diseases. Dietary supplements have beneficial effects on various risk factors associated with cardiovascular diseases. However, little is known about the role of probiotics, prebiotics and synbiotic supplements as important dietary components in reducing TMAO level. Our meta-analysis suggests that microbiota-driven therapy does not reduce level of TMAO and its associated metabolites. Our results are contrary to the study of Qui et al. (36), their experiment proved that L. plantarum ZDYO4 inhibited the development of TMAO-induced atherosclerosis in ApoE^−/−^ mice, compared to control. We speculate that part of the reason may be caused by the types of probiotics. However, due to the limitation of the number of literatures, we cannot perform subgroup analysis on the types of probiotics. In addition, some factors affecting TMAO level are unavoidable, such as renal function, age of patients and individualized diet. TMAO is cleared by the kidney, and prior studies have reported that the level of TMAO rises in subjects with impaired renal function (37). Mitchell et al. noted that seafood intake may interfere with their measurement of TMAO (38). Wang et al. found that low level of microbial-available carbohydrate diet may affect the gut microbiota and its activity, as well as the production of TMA (39). Further research, focusing on supplements that regulating the type of microbiota, different diets and different layers of renal function needs to be conducted, and large-scale clinical study is required to verify current conclusions.

### Strengths and Limitations

In this analysis, we summarized all RCTs assessing the effects of microbiota-driven therapy on circulating level of TMAO and its metabolites. The quality of the studies was high, and thus, the research conclusion was highly reliable. In addition, no significant publication bias was identified; therefore, to some extent, the influence of potential unpublished articles on the stability of the research results was excluded. Some limitations of this study should also be noted. First, the small sample size is undoubtedly a major limitation. Secondly, we were unable to explore in depth all the variables that theoretically affect TMAO level, such as diet, diabetes, kidney function, and concomitant cardiovascular medications.

## Conclusion

Current evidence does not support that microbiota-driven treatment reduce circulating level of TMAO, choline, betaine aldehyde, and L-carnitine. However, given the small sample size, this conclusion needs to be proved in the future.

## Data Availability Statement

The original contributions presented in the study are included in the article/Supplementary Material, further inquiries can be directed to the corresponding author/s.

## Author Contributions

HQ and DS: conceptualization and supervision. LM and HQ: data management, data analysis, methodology, software application, and writing (initial manuscript). JD and ZC: project administration. JD and LM: writing (review and editing). All authors contributed to the article and approved the submitted version.

## Conflict of Interest

The authors declare that the research was conducted in the absence of any commercial or financial relationships that could be construed as a potential conflict of interest.

## Publisher's Note

All claims expressed in this article are solely those of the authors and do not necessarily represent those of their affiliated organizations, or those of the publisher, the editors and the reviewers. Any product that may be evaluated in this article, or claim that may be made by its manufacturer, is not guaranteed or endorsed by the publisher.
