# Supplementary material for: Effects of Microbiota-Driven Therapy on Circulating Trimethylamine-N-Oxide Metabolism: A Systematic Review and Meta-Analysis
Source: Front Cardiovasc Med. 2021 Sep 6;8:710567. doi: 10.3389/fcvm.2021.710567 (PMC8450403; doi:10.3389/fcvm.2021.710567)
Supplement: Supplementary file 1 [file Data_Sheet_1.PDF]

Pubmed

#1 trimethylamine (Title/Abstract)

#2 trimethylamine N-oxide (Title/Abstract)

#3 TMA (Title/Abstract)

#4 TMAO (Title/Abstract)

#5 choline (Title/Abstract)

#6 Betaine (Title/Abstract)

#7 L-carnitine (Title/Abstract)

#8 #1or #2 or #3 or #4 or #5 or #6 or #7

#9 prebiotic (Title/Abstract)

#10 probiotic (Title/Abstract)

#11 synbiotic (Title/Abstract)

#12 dried yeast (Title/Abstract)

#13 lactobacillus (Title/Abstract)

#14 Bifidobacterium (Title/Abstract)

#15 Saccharomyces (Title/Abstract)

#16 probiotics in Clostridium difficile (Title/Abstract)

#17 L. GG (Title/Abstract)

#18 LGG (Title/Abstract)

#19 L. acidophilus (Title/Abstract)

#20 L. rhamnosus (Title/Abstract)

#21 L. plantarum (Title/Abstract)

#22 L. casei (Title/Abstract)

#23 L. gasseri (Title/Abstract)

#24 L. reuteri (Title/Abstract)

#25 L. lactis (Title/Abstract)

#26 B. breve (Title/Abstract)

#27 B. longum (Title/Abstract)

#28 B. infantis (Title/Abstract)

#29 B. adolescentis (Title/Abstract)

#30 B. lactis (Title/Abstract)

#31 Bacillus (Title/Abstract)

#32 Clostridium butyricum (Title/Abstract)

#33 Streptococcus thermophilus (Title/Abstract)

#34 Escherichia coli (Title/Abstract)

#35 Enterococcus SF68 (Title/Abstract)

#36 Enterococcus faecalis (Title/Abstract)

#37 Saccharomyces boulardi (Title/Abstract)

#38 VSL#3 (Title/Abstract)

#39 #9 or #10 or #11 or #12 or #13 or #14 or #15 or #16 or #17 or #18 or #19 or #20 or #21 or #22 or #23 or #24 or #25 or #26 or #27 or #28 or #29 or #30 or #31 or #32 or #33 or #34 or #35 or #36 or #37 or #38

#40 #8 and #39

EMBASE

#1 trimethylamine (ab, ti)

#2 trimethylamine N-oxide (ab, ti)

#3 TMA (ab, ti)

#4 TMAO (ab, ti)

#5 choline (ab, ti)

#6 Betaine (ab, ti)

#7 L-carnitine (ab, ti)

#8 #1or #2 or #3 or #4 or #5 or #6 or #7

#9 prebiotic (ab, ti)

#10 probiotic (ab, ti)

#11 synbiotic (ab, ti)

#12 dried yeast (ab, ti)

#13 lactobacillus (ab, ti)

#14 Bifidobacterium (ab, ti)

#15 Saccharomyces (ab, ti)

#16 probiotics in Clostridium difficile (ab, ti)

#17 L. GG (ab, ti)

#18 LGG (ab, ti)

#19 L. acidophilus (ab, ti)

#20 L. rhamnosus (ab, ti)

#21 L. plantarum (ab, ti)

#22 L. casei (ab, ti)

#23 L. gasseri (ab, ti)

#24 L. reuteri (ab, ti)

#25 L. lactis (ab, ti)

#26 B. breve (ab, ti)

#27 B. longum (ab, ti)

#28 B. infantis (ab, ti)

#29 B. adolescentis (ab, ti)

#30 B. lactis (ab, ti)

#31 Bacillus (ab, ti)

#32 Clostridium butyricum (ab, ti)

#33 Streptococcus thermophilus (ab, ti)

#34 Escherichia coli (ab, ti)

#35 Enterococcus SF68 (ab, ti)

#36 Enterococcus faecalis (ab, ti)

#37 Saccharomyces boulardi (ab, ti)

#38 VSL#3 (ab, ti)

#39 #8 and #38

Cochrane Library

#1 trimethylamine (ab, ti, kw)

#2 trimethylamine N-oxide (ab, ti, kw)

#3 TMA (ab, ti, kw)

#4 TMAO (ab, ti, kw)

#5 choline (ab, ti)

#6 Betaine (ab, ti, kw)

#7 L-carnitine (ab, ti, kw)

#8 #1or #2 or #3 or #4 or #5 or #6 or #7

#9 prebiotic (Mesh descriptor)

#10 probiotic (Mesh descriptor)

#11 synbiotic (Mesh descriptor)

#12 dried yeast (ab, ti, kw)

#13 lactobacillus (ab, ti, kw)

#14 Bifidobacterium (ab, ti, kw)

#15 Saccharomyces (ab, ti, kw)

#16 probiotics in Clostridium difficile (ab, ti, kw)

#17 L. GG (ab, ti, kw)

#18 LGG (ab, ti, kw)

#19 L. acidophilus (ab, ti, kw)

#20 L. rhamnosus (ab, ti, kw)

#21 L. plantarum (ab, ti, kw)

#22 L. casei (ab, ti, kw)

#23 L. gasseri (ab, ti, kw)

#24 L. reuteri (ab, ti, kw)

#25 L. lactis (ab, ti, kw)

#26 B. breve (ab, ti, kw)

#27 B. longum (ab, ti, kw)

#28 B. infantis (ab, ti, kw)

#29 B. adolescentis (ab, ti, kw)

#30 B. lactis (ab, ti, kw)

#31 Bacillus (ab, ti, kw)

#32 Clostridium butyricum (ab, ti, kw)

#33 Streptococcus thermophilus (ab, ti, kw)

#34 Escherichia coli (ab, ti, kw)

#35 Enterococcus SF68 (ab, ti, kw)

#36 Enterococcus faecalis (ab, ti, kw)

#37 Saccharomyces boulardi (ab, ti, kw)

#38 VSL#3 (ab, ti, kw)

#39 #8 and #38
